# Operative Dentistry

**Published:** 1895-04

**Authors:** 


### MONTHLY DIGEST.

### Operative Dentistry.

(*Continued from Page* 148.)

### THE WILSON CROWN.

DR. H. H. WILSON* describes a crown for which he claims two points of advantage—increased strength, and ease of construction. The tooth to be crowned is cut off a little below the gum line, a narrow band of 22 k. gold is fitted flush with the end of the root and a flat piece of 30 or 32 guage gold soldered to it, forming a cap. A suitable Logan crown is selected and the palatal portion ground away without disturbing the pin. With the cap in place on the root, a hole is pierced in it, the post of the crown inserted and correct position obtained. The crown is then removed, the cap being left in position, and the post forced through a second disk of the same gold large enough to more than cover the ground surface of the crown. The gold is burnished down and trimmed off. Sticky-wax being placed around the pin, it is once more placed in position, all parts removed together, inverted and soldered. This crown can be completed at one sitting. Gutta-percha is preferred for setting.

### LOCAL EXERCISE AND DIETETIC INFLUENCE UPON THE TEETH.

Dr. S. S. STOWELL read a paper on this subject before the First District Dental Society of New York.†

The prime factors in the laws of life and health are *exercise* and *diet*. When the normal use of an organ is discontinued, nature proceeds to eliminate it, the human teeth being no exception to this rule. *Local exercise* is of the first, and *diet* of secondary importance in the salvation of the teeth. Many cases were cited by the essayist in illustration of the position that lack of local

* Dental Review, December 15, 1894.
† Cosmos, January, 1895.

exercise is the prime factor in the retrograde metamorphosis of the human teeth of the present age. The remedy is simple; subsist upon food that requires masticating, that the teeth may have exercise like the other organs of the body, and they will develop strength and health. In the discussion of this paper, the ground was taken by Dr. Jarvie, Dr. S. G. Perry and others, that the present generation is better physically than the people of several generations back; that this is an age of athletic exercises; that our present mode of living has not degenerated, etc.

Dr. W. Irving Thayer emphasized the two points made by the essayist—that what is wanted for the teeth is not athletic exercises in general, but special local exercise for the teeth, and proper pabulum for the stomach and the teeth.

### ETIOLOGY OF DEFECTIVE ENAMEL.

This is the subject of a paper by Dr. W. E. Royce, read before the American Dental Society of Europe.*

After a brief *resume* of the most important points in the development of the teeth, Dr. Royce reaches the conclusion that while some of the lesions referred to are due to prenatal influences, a great many are caused subsequent to birth, largely due to stomatitis from the use of mercury, attributing to hereditary mercurialization much of what Hutchinson attributes to hereditary syphilis—this through the direct action of the poison upon the germinal cells, as is transmitted alcoholic poisoning. In proof of his position, he exhibited the skulls of puppies treated with mercury, in his repeated experiments three puppies from the same litter having been selected, of which one was treated with mercury, another with Grey powder, the other not treated. These experiments will be continued upon rabbits.

### PATHOLOGICAL CONDITION OF THE MUCOUS GLANDS OF THE MOUTH.

Dr. Geo. H. Winkler read a paper on this subject before the Homeopathic Medical Society of the County of New York,†

* Dental Digest, January, 1895.

† Cosmos, January, 1895.

discussing causes, appearance, results and treatment. The writer believes the condition to be due to "elimination by these glands of hereditary poisons," the glands themselves becoming poisoned, causing severe inflammation and sloughing, with most disastrous results, not only in the mouth, but sometimes in the stomach. Of the treatment he said: "I am an allopath both by medical and dental education, and for years exhausted the resources of that school in my efforts to combat this condition. . . . The paucity of internal remedies furnished by allopathy for the treatment of diseases of the mouth, and the accidental reading of a homeopathic paper, led me into a study of the homeopathic treatment of the mouth and the remedies. Among the latter I found *kreosotum*, which, in my four years use of it, has proved itself to be almost an absolute specific for the disease we are considering," (acrid, corrosive, slimy secretions, rapid white decay, erosion, etc.)

### THE FINAL REPORT OF DR. J. J. R. PATRICK ON THE EXAMINATION OF PREHISTORIC CRANIA,

Under the auspices of the American Dental Association, is given in the *Cosmos*,* with the editorial comment: . . . "The dental profession may well be congratulated that this important collection of data has been placed in such shape that it may be utilized for further study and investigation. Such work is a necessary preliminary to all truly scientific study." . . .

### TEMPERAMENTS.

Dr. Geo. B. Perry, in a paper read before the Chicago Dental Society†, after quoting from various authorities, gives his personal experience with a few cases, illustrating the four temperaments as usually accepted, and also some of the idiosyncracies and peculiarities met with in the dentist's daily practice. The discussion of the paper ran upon character and disposition rather than temperaments *per se*.

### HYPNOTISM.

Dr. H. H. Fitch, in a paper read before the First District Dental Society of Illinois,‡ reviews the subject of hypnotism,

*Dental Cosmos, December 15, 1894.
†Dental Review, January 1895.
‡Dental Review, December 15, 1894.

quoting numerous authorities, showing its advantages as an anæsthetic, and the objections to its use, these being chiefly: the time necessary, the small percentage susceptible to hypnosis, the exhaustion of nerve force on the part of the operator, the vagueness as to the methods of dehypnotizing, the dangers of "suggestion" in weakening the patient's power of self-control, and the danger arising from the popular prejudices which would attribute any subsequent moral or mental obliquity to the effect of previous hypnosis.

The *International Dental Journal* publishes another section of the Address (to be continued) of Dr. A. H. Porter before the Alumni Society of the Department of Dentistry, University of Pennsylvania, on "The Physiology of Hypnotism."

MODERN ANÆSTHESIA.

The memorial celebration of the fiftieth anniversary of the discovery of anæsthesia, December 11, 1894, naturally occupies much space in the January journals.

The *International Dental Journal*, the *Dental Cosmos*, and the *Dental Digest* give abstracts of the address of Dr. Thos. Fillebrown, in which he presents "the history of the development of anæsthesia, from the earliest periods of human history until it culminated in the final discovery by Wells." Also of the address of Dr. Jas. E. Garretson, who took "the philosophical and humanitarian side of the question."

The toasts at the banquet were responded to as follows: "The Horace Wells Discovery," by United States Senator General Joseph B. Hawley of Connecticut; "Anæsthesia as a Dental Discovery," by Dr. James Truman; "Anæsthesia as a Factor in the Evolution of Surgery," by Dr. J. William White, University of Pennsylvania; "The Mastery of Pain from the Standpoint of the Layman," by Col. Alexander McClure, editor-in-chief of the Philadelphia *Times*; "The Development of Our Knowledge of Anæsthesia," by Dr. Wilbur F. Litch; "The Medico-Legal Aspect of Anæsthesia," by District Attorney Geo. S. Graham, o Philadelphia; "The Humanitarian Aspect of Anæsthesia," by Rev. S. D. McConnell, of Philadelphia. Dr. G. Q. Colton, who administered the nitrous oxide on that memorable occasion fifty years ago, detailed the facts as he knew them, and Mr. Charles Wells, son of the great discoverer, paid a feeling tribute to the memory of his father.

The occasion is characterized by the *International Journal* as "one of those epoch-making periods that come seldom in the history of professions and peoples, but when they do appear they mark a dividing line between the old and the new."

An account of the Hartford celebration of the event,* found in the *Cosmos*, is illustrated with a *fac simile* of the memorial tablet erected in honor of Horace Wells, unveiled on the occasion.

### SCIENTIFIC WRITERS.

Editorially the *Review* criticises an apparent lack in the writing of scientific men, *viz:* that they draw no deductions of practical application from their work. The average reader is either not capable of intelligently drawing deductions, or will not take the trouble to do so.

For scientific investigations to benefit dentistry, scientific articles must be read, and to be read they must be carried down to a practical application. This would not, in any degree, interfere with a purely scientific habit of thought, and would immeasurably extend the usefulness of the work of scientific men.

### DENTAL EDUCATION.

Dr. P. D. Houston, in a paper read before a general meeting of the Nashville Academy of Medicine,† discusses the subject of dental education, especially that phase concerning patients and the public—those in need of dental services but in ignorance of the capabilities of dentistry, suggesting the propriety of a text-book, simple, concise and cheap, for use in our public schools as the most efficient means of educating the people.

Dr. W. J. Morrison read a paper before the dental section of the same society‡ in which he urged the importance of impressing upon students the value of the teeth in their relation to the

*Dental Cosmos, January, 1895.
†Dental Headlight, January and March, 1895.
‡Dental Headlight, January and March, 1895.

rest of the human system and their function in digestion, also the necessity of instructing the public in the care of the teeth and the hygiene of the mouth. He dwelt especially upon the injury done to the human race by the injudicious extraction of teeth. While regretting the large percentage of men in the profession who pride themselves on their skill in the extraction of teeth, he said: "I would not be understood as deprecating the skill of an operator in performing the operation of extracting when necessary, any more than I would disparage that of a gynecologist in sacrificing the child to save the mother when conditions require."

DR. W. F. FOWLER, in a paper read before the East Tennessee Dental Association* reviews the "Rise and Progress of Dentistry" along the lines of education and literature, methods, materials and appliances, inventions and discoveries.

DR. J. C. WALTON, in a paper bearing the title "Popular Dental Education and the Newspaper,"† and with the phrase from Article IV of the Code:

"It is our duty to enlighten and warn the people" as a text, says: "There is little humanity and less philanthropy in withholding beneficial knowledge where innocent ignorance is of necessity the greatest sufferer. With seventeen thousand dentists in the United States, many of whom are desperately idle, and yet with dentistry enough that should be done to keep fifty thousand dentists busy, some way should be found of making the needs of the people and the needs of the dentists fit into each other. The remedy for this condition of things lies in the educa tion of the public, both as to their dental needs and as to the proper source of service. Commercialism has thus far made the most successful appeal to the masses. Dignified, uncompromising silence does not enlighten the public; the "pamphlet" system has not proved effective; the newspaper remains as the resource needed for popularizing effective dental teaching. Being acceptable to the masses, it is the surest, quickest and most economical way of reaching the public."

---

* Dental Headlight, January and March, 1895.

† Dental Cosmos, February, 1895.

The essayist concludes with the outline of a plan which appears to be liberal, just, ethical and philanthropic, and which might be made effective and profitable.

SYSTEMIC DENTAL TREATMENT.

In the discussion by the New Jersey State Dental Society,* of the paper on "Systemic Dental Treatment,"† by Dr. L. Ashley Faught, Dr. Sanger said: "If we will only feel that we have the right, and will take the right, to practice medicine when the practice of medicine is indicated in dealing with our patients, having first thoroughly fitted ourselves to do so, then I believe we shall have . . come upon a higher plane, and that we will be better able to do for our patients the good that they expect and desire at our hands."

Dr. J. Foster Flagg said that, after listening to the paper, he felt that he had been derelict in his duty in not speaking more to that point in his lectures from the chair of pathology and therapeutics, and that in the future he should place legitimate medicament in a very much more prominent position, and strive to induct the idea of appropriate systemic treatment in combination with dental treatment.

In continuation of the same line of thought, Dr. Faught read a paper on "Dental Therapeutics" before the First District Dental Society of New York,‡ expressing the opinion that the rapid development of the dental profession is most prominent in the line of dental therapeutics, both local and systemic; that the dental practitioner of to-day who is not acquiring an ever-increasing knowledge of medicaments and their uses, is not capable of giving his patients that intelligent aid that they have a right to expect. While it is a duty to repair the ravages of dental disease, it is a higher duty to prevent this manifestation of disease and to alleviate suffering—not alone, however, by the administration of drugs—"the gospel of fresh air, sunshine and out-door exercise is the sermon that must be continually upon our

* Dental Cosmos, February, 1895.

† Dental Cosmos, December, 1894.

‡ Dental Cosmos, February, 1895

tongues." Having, in the previous paper, given a list of some thirty remedies constituting a basis for systemic dental medicine, the present paper is directed more especially to the influence of the nervous system on the health of the teeth and mouth, and the therapeutic treatment necessary in the restoration of "tranquility of nerve," to the impairment of which dental diseases are mainly due. The therapeutic remedies directed towards the organs of excretion, and especially the uric acid soluents, are the special topic of this paper.

In the discussion, Dr. E. C. KIRK questioned whether it would not be infringing upon the field of the general practitioner to follow out the principles laid down by Dr. Faught. While agreeing that every dental practitioner should be sufficiently intelligent and sufficiently educated to diagnose the systemic causes of dental disorders, he thought that in doing that he had gone far enough. To undertake the systemic treatment would be as though the physician, on finding that certain systemic conditions were due to diseased conditions of the teeth, should proceed to treat and fill the teeth!

DR. VAN WOERT agrees with Dr. Kirk, that it is not within the province of the dental practitioner to go beyond the local treatment. In closing the discussion, Dr. Faught said that the argument of his paper was not so much to advise entering into the treatment of the diseases or the use of drugs, as to lead our patients into hygienic lives, so that by going back of the trouble we can quiet that nerve trouble which causes the pain.

### HYPNOTISM.

In a paper entitled the "Present Scientific Status of Hypnotism,"* Dr. W. X. Sudduth claims that hypnotism is one of the most common forces in nature; that we are subject to its influence at all times, and that were it not for *suggestion*, which forms the basis of hypnotism, we could make but very little progress, being, as we are, creatures of environment, dependent upon the labors and suggestions of others in most things. He finds a familiar illustration in its constant and harmless use in childhood; every

* Dental Review, January 15, 1894.

time that a mother or nurse puts a child to sleep, she hypnotizes, the means being the monotonous swing of the cradle, the lullaby song, the reiterated "go to sleep, sleep, sleep," etc. When a similar moral atmosphere can be established around the adult, the greatest benefit will be obtained from suggestive therapeutics.

Dr. Sudduth holds, with Bernheim, that hypnotism is closely allied to natural sleep, and, like the latter, can be made a valuable therapeutic agent in the alleviation of many physical disorders. It is only within the past few years that the subject has been placed upon anything like a scientific basis, but with the advance in our knowledge regarding the physiology of function through physiological chemistry, we are becoming better prepared to direct it. We have caught only a glimpse of the boundless possibilities of suggestive therapeutics coupled with a judicious use of specific drugs.

Quoting from Bernheim, he says: "The patient is put to sleep by means of suggestion—that is, by making the idea of sleep penetrate the mind. He is treated by means of suggestion—that is, by making the idea of cure penetrate the mind. . . . We profit by the special psychical receptivity created by hypnosis, . . . to persuade the brain to do what it can to transform the accepted idea into reality." After enumerating the various hospitals that, either exclusively or in certain wards only, are treating and curing functional disorders and organic disease by hypnotic treatment, and quoting a long list of cases (several pages), successfully treated by suggestive therapeutics, he says: "The special line of work that I have been, and am still engaged upon, is its application to surgery," having in his own clinics daily demonstrated its value as an anæsthetic, and concludes: "It is fitting that a dental clinic should be the first to take the lead in the matter, as dentistry has always been in the foremost rank in experimenting with anæsthetics, and with excellent results. Dentistry gave nitrous oxide gas and ether to surgery, and now leads in the introduction of hypnosis."

The paper was discussed at some length, Drs. Bailey, Weeks and Dickinson making a distinction between "suggestion" and "hypnotism," and opposing the use of the latter in dental practice.

Drs. Knight and Wood cited several cases of what might be the unconscious exercise of this power by the operator.

Dr. H. C. Aldrich, a practicing physician, is a firm believer in hypnotism and the good to be obtained from it.

Dr. M. B. Wood does not believe the possibilities for harm greater than from the use of drugs. Dr. F. E. Twitchell thinks the dentist should do all in his power to relieve pain, and does not believe in "leaving to the devil all good things." Dr. Sudduth, in closing the discussion, emphasized the necessity for the use of the same safeguards in the practice of hypnotism as in the administration of other anæsthetics, giving the following rules: "Never administer any anæsthetic to a woman without a witness who is *your* witness; never practice hypnotism without the presence of a witness who is a friend of the patient, and a witness *also* who is *your* witness. Under these conditions, practice it. Use it the same as any other powerful remedy."

At the Union Convention in Buffalo, N. Y.,* Dr. W. W. Belcher read a paper on "The Relation of Nitrous Oxid and Asphyxia, reaching the conclusion that nitrous oxid is asphyxiating, and to be used with caution, although the safest agent in use for producing unconsciousness; that no man has the right to jeopardize the life of a fellow-being for a minor operation such as the extraction of a single tooth.

During the discussion, Dr. George Fell demonstrated the use of his forced respiration apparatus.

### HYDROGEN DIOXIDE.

Dr. Henry Leffmann, in a paper read before the Odontological Society of Pennsylvania,† furnishes the results of tests made during the past year, of samples of the hydrogen dioxide solutions sold to dentists, including samples from S. S. White Manufacturing Company, Sibley and Justi, the tests being both for acidity and for volumes of oxygen. The samples were in original packages, and were used when fresh. The figures given show very inferior samples, one-ounce bottles from the three

* Dental Cosmos, February, 1895.

† International Dental Journal, February, 1895.

houses named, tested in February, 1894, showing, respectively, S. S. W., 5.1 and 5.4; Sibley, 0.5; Justi, 1.4. McKesson & Robbins' pyrozone, opened occasionally during six months that it was standing in the laboratory, showed 9.1, containing when fresh almost exactly 10 volumes. Dr. Leffmann adds: "There is no reason why the large supply houses should retail such inferior samples as above noted."

In the discussion, he said: "A sample of English hydrogen peroxide, which I examined, costs one dollar for a four-ounce bottle and contains only about six volumes of active oxygen. How imperfect must the treatment be that depends upon such inferior articles."

In reply to a question regarding the tests for acidity, he said: "The acidity in Marchand's peroxide amounted to about eight in the test made to-day, against about four—the minimum—in the Oakland. The acidity of hydrogen is about two for the same volume."

### AN ELECTRICAL DISINFECTANT.

Mr. Albert E. Woolf, New York, read a paper before the Central Dental Association of Northern New Jersey,* giving the results of a series of experiments on the action of the electrical current on sea water, obtaining from the decomposed sea water an electrical disinfectant —"Electrozone"—having a most destructive disintegrating power upon germs and germ life, with the advantage of being perfectly harmless—absolutely non-poisonous. As manufactured for commercial use for disinfecting on a large scale, putrid water and poisoned sewage are rendered innocuous, decomposed garbage is freed from all offensive odors, putrid meat in an advanced stage of decomposition is made sweet, etc.

Numerous experiments were made before the society upon various forms of albumen (including "ancient eggs"), illustrating the powerful action of the disinfectant.

The grade of this disinfectant suited for dental and medicinal use has not as yet been manufactured, except in experimental quantities, and is not on the market, though samples will be supplied for experimentation through Dr. Geo. C. Brown.

* International Dental Journal, February, 1895.

### ABSCESS OF THE ANTRUM OF HIGMORE.

Under this title Dr. E. S. HODGSKIN, M. D., treats of the causes of this disease and the symptoms in both acute and chronic cases, the diagnosis and the treatment, in a paper read before the Second District Dental Society of New York.*

As an antiseptic Dr. Hodgskin prefers peroxid of hydrogen, with the precautionary measure of first washing the pus out of the cavity with distilled water to avoid the formation of too much gas, with consequent distension and pain.

In the discussion Dr. V. E. Houghton said that he used only warm water and chlorid of zinc, being afraid of the effervescence of peroxid of hydrogen.

In the experience of Dr. W. E. Halsey, Listerine—one to twenty parts of water—meets every indication.

Dr. R. H. Sturgis M. D., expresses the supposition that in some cases the teeth may be affected secondarily, as the result, rather than the cause, of empyæma of the antrum.

### PYORRHŒ ALVEOLARIS.

At the September meeting of the Minnesota State Dental Association,† Dr. W. X. SUDDUTH read a paper in continuation of the one read at the May meeting of the same society, published in the June and July, 1894, issues of the *Dental Cosmos.*

Accepting the simpler forms of the disease as an acute catarrhal process, easily amenable to palliative medication, combined with such mechanical handling as the case in hand may indicate, Dr. Sudduth gives minute details of treatment, with a long list of prescriptions for local applications or internal administration, designed to meet the varying conditions encountered—acid conditions, catarrhal conditions, the suppurative form, constitutional complications, the gouty diathesis, neuralgia, etc. In suppurative catarrhal conditions Dr. Sudduth lays special stress upon the value of sozo-iodol and zinc, to which he has called attention frequently since 1888, when he first directed the attention of the profession to its merits. As an appendix to the

* Dental Cosmos, February, 1895.

† Dental Cosmos, February, 1895.

article there is given an extended bibliography of the drugs and literature of Pyorrhœ Alveolaris.

### THE ETIOLOGY OF PUS FORMATION.

Dr. S. Eschelman read a paper bearing this title before the Union Convention, in Buffalo, N. Y.*

He considers the phenomena of inflammation as but the exalted, or rather perverted, phenomena of normal nutrition. The dilatation of the capillaries, wandering out of the white corpuscles, effusion of liquor sanguinis, destruction and regeneration of cell life—the physiological means of carrying on the normal functions of the body—may from repeated irritation become pathological. The function of the cells being disturbed, if the irritation is light it may lead only to hyperplasia, without inflammation, as in the thickening of the epithelium on the hands of the laborer, etc. If the irritation be more pronounced, as in traumatic injury, inflammation may be developed, progressing through the suppurative stages to the production of pus, unless kept in an aseptic condition, thus excluding secondary causes.

An injury is not in itself sufficient to develop the inflammatory action to the production of pus. The argument of the paper leads to the conclusion that pathogenic bacteria are the essential and direct cause of suppuration, and that by their growth, development and formed ptomaines, they are capable of initiating the inflammatory phenomena and developing them successively through all the stages to the production of pus.

### THE TREATMENT OF PULPLESS TEETH.

Dr. F. T. Van Woert, in a short paper,† gives the details necessary for the accomplishment of the best results in the use of peroxide of sodium, and sulphuric acid, in root canals.

Having used the latter exactly by the methods described by Dr. Callahan, his experience fully justifies the claims made for it, making it possible to master many otherwise hopeless cases.

Great care and ample time are required for the preparation of the sodium peroxide solution, but when properly made it can

* Dental Cosmos, February, 1895.

† International Dental Journal, February, 1895.

be kept in a glass-stoppered bottle, in a cool place, for a long time. Dr. Van Woert gives the following directions for its preparation: "Take a common tumbler about half full of distilled water, place it in the center of a good-sized pudding dish and pour all the cold water around it possible without floating the glass; add the sodium peroxide in very small portions, about what could be taken upon the point of the large blade of a pocket knife, dusting it in the water slowly to cause as little agitation as possible, and this amount should not be added oftener than once in a half hour, being careful to have the sodium peroxide finely powdered; this to be continued until the preparation begins to look opaque as powder is added. Let it stand over night, and it is then ready for use."

Dr. Van Woert said that he had received from a student in the Philadelphia College reports of very successful results from placing a small portion of the powdered peroxide in the pulp chamber, flooding the cavity with water, and allowing it to remain until the agitation from the combination ceases, after it is washed out and treated as before.

He adds: "This would seem to me a very practical and sure way of obtaining a solution that would be effectual."

In the discussion, by the New Jersey Dental Society,* of Dr. C. N. Johnson's paper on "The Management of Pulpless Teeth" (*Cosmos*, Dec., 1894), Dr. Peirce spoke favorably of the use of cobalt for pulp devitalization, as by its use there is less liability of getting too much arsenic in the tooth, and it can be left in long enough to insure removal of the pulp without tearing it in pieces. He also spoke favorably of the use of cotton in root-canal filling, the cotton being treated with aristol moistened with chloroform, and pressed to the end of the root, the rest of the canal being filled with gutta-percha.

Dr. Wm. H. Truman grinds arsenic to a paste with creosote and a little glycosal. He is not particular about the amount and not believing there is any risk of its passing beyond the apex of the root in ordinary cases. He recommends the use of cajeput as a lubricant when filling with gutta-percha.

* Dental Cosmos, February, 1895.

Dr. Watkins considers cajeput used in this way a complete failure, being a great irritant. He uses eucalyptol with chloro-percha. He always uses iodoform in the canal and fills without removing it.

Dr. Jas. Truman protests against the statements made as to the use of indefinite amounts of arsenic left in the tooth for an indefinite time. He considers arsenic unlimited in its destructive power, passing through the foramen with resulting necrosis of the parts reached. He wants exact figures and believes the thousandth part of a grain of arsenic sufficient to carry destruction to the upper third of the canal. If it goes beyond that it endangers the pericementum.

Of *curing abscesses*, he said: "In my opinion there has never been an abscess cured, and there never can be, unless the cause of the abscess is removed. From my experience I don't believe there is a single particle of live tissue at the end of a root where there has been alveolar abscess."

"An abscess means the destruction or suppuration of the pericementum on the root of the tooth, and that once removed, there can never be a return of vitality, and that particular part of the tooth must be inevitably necrosed. How are you going to cure it? You can temporarily relieve it, you can close up the fistula, but beyond that you can not go, unless you remove the necrosed portion."

Dr. J. Foster Flagg, in advocating the use of cotton in filling the root canals, wants "natural cotton with its natural oil"—not "absorbent cotton." He said: "The fact is, gentlemen, a cotton filling can be made as perfect as any filling, and as non-leaking as any filling can be made."

### PUTRESCENT PULP CANALS.

Dr. D. W. Barker gave the New York Odontological Society* an improved method of disinfecting putrescent pulp canals, which has not the objection of causticity found in other methods now in use. By bringing into contact, in the root canal, permanganate of potash and peroxide of hydrogen, violent effer-

* International Dental Journal, February, 1895.

vescence takes place with the liberation of nascent oxygen. The powdered permanganate of potash is introduced into the canal on a Donaldson bristle, peroxide of hydrogen dropped on it with a syringe, and the bristle quickly pumped up and out. The teeth so treated show remarkably quick cure.

In reply to a question, Dr. Barker stated that he had not known it to produce any irritation of the periosteum.

ROOT CANAL FILLING.

Dr. L. W. Skidmore, in a paper read before the First District Dental Society of Illinois,* presents a review of the various method and materials in use for root-filling, and gives his own methods of treating "pulpless teeth with putrescent pulps." After the removal of all debris and drying with cotton until there is no trace of putrescent matter, the canals are washed with peroxide of hydrogen or pyrozone, followed by alcohol, and the canals dried with hot air. A dressing of "cassia 1, enymol 2, galelthesia 3," is then applied on cotton and left in for several days. When the roots are perfectly sweet with no soreness remaining, the canals are moistened with eucalyptol, to assist the passage of chloro-percha, or gutta-percha and eucalyptol, which is worked thoroughly into the canals and followed by gutta-percha cones. A roll of vulcanite rubber is then placed in the cavity and with a blunt instrument pressed into the chloro-percha forcing it into all parts of the canal which may then reasonably be considered thoroughly filled.

At the December meeting of the Second District Dental Society of New York† the question: "Do the Oxyphosphates and the Oxychlorides, When Placed in Contact, or in Close Proximity to the Pulp, Cause Its Death?" was introduced for discussion by Dr. J. P. Geran, his own opinion being that they will eventually destroy the pulp unless there is a thick intervening section of good healthy dentine between the pulp and the cement.

Dr. F. E. Howard believes that death of the pulp will

*Dental Review, January 15, 1895.

† Dental Cosmos, February, 1895.

follow the use of the oxyphosphates when sterilization of the cavity with a coagulating antiseptic has been neglected. The addition of creosote—combining enough with the paste before it begins to set to render it permanently antiseptic—so modifies the action of the oxphosphate that he uses it with impunity provided that the pulp is protected by a thin covering.

A true pulp-capping material should possess both antiseptic and stimulating properties to induce an effort toward recuperation, in the deposition of ossific material, the natural protector of the pulp.

Dr. S. B. Palmer explained the death of the pulp under thin cappings as due to the acid set free by the decomposition of the cement by moisture from dentine containing much organic matter. The moisture in the dentine having an affinity for the acid of the cement, the dentine becomes inflamed and the lime element is taken back by nature. When the dentine is normal, and the pulp protected—as by varnish—the pulp is not destroyed.

Dr. J. Foster Flagg excludes the oxychloride of zinc because properly used only as a lining for the walls of cavities in teeth less than "above medium" in structure, and always preceded by red base-plate, rubber varnish, temporary stopping, or zinc sulphate.

Zinc phosphate he would not, as a rule, place in any doubtful contiguity to vital pulps, although in many instances large cavities, closely approaching vital pulps, have been satisfactorily and successfully filled with it.

Dr. W. S. Elliott thinks that when success follows this use of oxyphosphate it is due to the previous use of an escharotic—preferably Merck's creosote, and never carbolic acid. This arrests the function of the peripheral cells of the pulp tissue though leaving them the power of depositing lime-salts in an amorphous condition.

Dr. D. W. Barker uses a combination of iodoform, or aristol mixed with the cement liquid, applied in a thin layer when the pulp is almost exposed. He also uses with success a small piece of court-plaster applied over the region of near exposure.

DR. C. S. VAN ORDEN places in the bottom of the cavity a thin piece of asbestos paper with Canada balsam, and the cement over that.

### THE MINERAL CEMENT.

PROF. S. J. WILLEY* outlines the principles governing the preparation of the materials entering into the cements and the methods employed in their manufacture, shows the fallacy of the popular idea that they can be easily, cheaply and *successfully* made by the dentist from the crude ingredients purchased at any drug store, and gives, as the result of a long series of experiments and consultations with a number of the best operators, valuable suggestions as to the correct method of mixing the mineral cements.

### AMALGAM WASTE.

DR. WM. H. TRUMAN† shows that the only really economical method of utilizing amalgam waste is to reduce it to an alloy by remelting to expel the mercury, casting into an ingot, and reducing to filings.

Attempts to save the mercury involve too much risk and expense though interesting as a laboratory experiment. At the present commercial valuation of silver any attempt to refine amalgam waste will be a financial disappointment while the proportion of gold in the alloys now on the market is too small to pay for the cost of refining.

### CARE OF CHILDREN'S TEETH.

DR. N. A. STANLEY read a paper bearing this title before the Harvard Odontological Society.‡ He urges that every possible effort be made to preserve the first teeth until the permanent teeth erupt according to natures' plan which cannot be improved upon. He enumerates the probable ill effects of too early extraction, and the good results of preserving the first teeth in presenting a good surface for proper mastication and allowing

* Items of Interest, February, 1895.
† Items of Interest, February, 1895.
‡ International Journal, February, 1895.

of full development of the jaws, also in holding back the permanent teeth till full time for their eruption with fully matured normal structure; another important benefit is the habit, early formed, of taking proper care of the teeth. An important duty of the dentist is to so instruct patients that future generations may inherit a dental structure less prone to disease. He believes that the best results will follow the use of lime-water by both mother and child.

The treatment of very young patients must be left to the judgment of each operator, but their confidence and friendship must be won and care taken not to frighten, fatigue or hurt them in their early experience at the dentist's hands.

In approximal cavities in the first teeth he uses cement. For crown cavities, if not deep, amalgam; otherwise, cement or gutta-percha, not attempting very thorough excavation. For buccal cavities and those at the margin of the gum he uses gutta-percha. In corono-distal cavities, amalgam at the cervical wall, and cement for the rest of the filling. If a pulp is exposed he destroys it with arsenic, and after treatment with warm water followed by three per cent. pyrozone, fills the pulp-cavity with iodoform paste, or cotton, finishing with cement. When extraction is inevitable he uses chloride of ethyl, but not spraying until white.

In the discussion of the paper, Dr. Warner and Dr. Bailey dissented from the opinion of the essayist that premature extraction of the deciduous teeth would hasten the eruption of the permanent teeth while imperfect in structure.

Dr. Smith is opposed to the use of amalgam in the temporary teeth believing that it causes death of the pulp.

Dr. Grant commends Dr. Pierce's method of applying nitrate of silver on little pads of blotting paper.

Dr. C. H. Taft removes the discoloration caused by nitrate of silver by applying iodine and a solution of salt and water, followed by cyanide of potassium. He spoke of the possibilities of the prevention of decay by constitutional treatment, using the homœpoathic remedies to supply the inorganic substances that enter into tooth structure; such remedies intelligently prescribed would help to prevent the necessity for surgical treatment.

It would appear from the discussion that it is rarely deemed necessary to fill cavities in the deciduous incisors.

IMPLANTATION.

Under the head of "Incidents of Office Practice," the subject of "Implantation" was briefly discussed by the New York Odontological Society.*

Dr. Jarvie gave the history of a case in which absorption of the root of a permanent right central incisor had taken place on both labial and palatine sides of the root, reaching into the pulp-canal. The tooth was free from decay, but the severe pain complained of by the patient led him to drill into the palatine surface for the purpose of killing the pulp. In treating the canal, after removal of the pulp, oozing out of the medicaments led to the discovery of the absorption. The tooth was treated and filled, but subsequently broke off at the point weakened by the absorption. It was decided to remove the root and replace the tooth by transplantation. In order to extract the frail root without bruising the gum or injuring the socket, he drilled through the center of the root with a Gates-Glidden drill, cut a thread in this and inserted a screw, firmly pulling out the root without any injury to the parts. Not being able to find a suitable natural tooth, he selected a good, suitably-shaped natural root, to which he attached a porcelain crown matching the natural tooth perfectly. The operation is too recent to pronounce upon the result.

The *periosteum* question being raised, Dr. Jarvie said that though he preferred to use a tooth with the periosteum intact, he would not say that it is essential. Dr. Perry thinks it does not make much difference, the age of the tooth to be implanted being the most important point; old, hard teeth, being the most favorable. He does not think a close fit in the socket essential, though it is valuable in the beginning.

Dr. Jarvie prefers to have it not fit too tightly in the newly-made socket. He cited a case in which the socket, after removal of diseased tissue, was nearly twice as large as the root of the tooth which he put in, ligating it to the adjoining teeth.

* International Dental Journal, February, 1895.

That was seven or eight years ago, and the tooth is now the firmest one in the mouth (the others, however, being rather shaky from pyorrhœa alveolaris).

Dr. Perry allows teeth saved for implantation to dry up, soaking them in bichlorid for a short time before using.

Of over a hundred cases of implantation, not more than one-half are probably in to-day; but he thinks that more than one-quarter are handsome successes. Often when an implanted tooth comes out, he slips in another—in one case four times in the same socket, in a period of six or seven years.

### PORCELAIN INLAYS.

Dr. A. W. Harlan read a paper on this subject before the Chicago Dental Society. In the discussion of the paper,* Dr. Wassall said that he had found this work very unsatisfactory for front teeth, because of the difficulty of matching the color, and also of subsequent changes in the color. On the buccal surfaces of molars he has found inlays more serviceable than gold fillings.

Dr. A. E. Mattison has experimented for a number of years, but finds that he can take more time to make an inlay than to make a continuous set of teeth, and when finished it is not satisfactory.

Dr. A. C. Hewitt has obtained very satisfactory results, some of his inlays having stood the test of ten years' service without any perceptable deterioration.

Dr. W. G. Taggart thinks that the change in color results from not perfectly fusing the porcelain. He believes that when the electrical furnace comes into use, there will be no further trouble in baking porcelain to the color desired. Instead of baking porcelain inlays by the present methods, he would use the English teeth, selecting exactly the color and shading required; mounting the section on a mandril, grind to approximate size, and then, with lots of water, grind it into the cavity with its own grit. These inlays should be inserted with "jeweler's shellac," which has a sticky quality different from pure shellac. It is called "Bottom's wax."

* Dental Review, January 15, 1895.

### CROWNS.

Dr. D. M. Gallie read a paper before the Wisconsin State Dental Society,* entitled "When and How Should Teeth be Crowned?" He said that he emphasized the word TEETH in contradistinction to ROOTS "because in this day of dental abortionists we find teeth cut down, ground down, broken off, and in every way sacrificed, for the sake of substituting a dental article called *a crown*, but which in many cases more resembles miniature tomato cans and sections of stove pipe" (!) the remainder of the profession being "compelled to do sewerage work" for these "self-magnified experts" in the removal of these "filth-traps and catch-basins of oral sewage." Dr. Gallie thinks that fully fifty per cent. of the teeth that are crowned to-day could be successfully filled, and that it would often be better to fill, even at a little risk as to the number of years the filling may last; should it subsequently fail, we still have a good foundation for a crown as the last resource. The natural crown should be substituted by an artificial one only (1) when the original is so badly decayed that its frail walls can not be strengthened sufficiently with cement to retain a permanent filling; (2), when the tooth structure is so soft and frail that it will fracture under the pressure to insert gold; (3), when the teeth are so malformed and irregular that crowning will be more advantageous than regulating; (4), when the tooth is badly discolored and the enamel checked, indicating that if bleached it would discolor again; (5), when a sufficient number of teeth are out to warrant the cutting down of two for the attachment of a bridge; even for the latter purpose, Dr. Gallie would not cut down two good teeth for the insertion of a dummy between, neither would he grind down and crown a good tooth to attach a dummy to it. Other negative cases are pointed out, and having thus narrowed the limits of *When* to crown, he proceeds to the question *How* to crown. The first point is always to devitalize the pulps, if not already destroyed. The paper then deals with the preparation of the root, according to the character of the crown to be used; the adjustment of the band to avoid

* Dental Review, January 15, 1895.

irritation of the gum, etc., with a description of Dr. Taggart's method of using the "Diatoric Tooth in Crown and Bridgework."

A backing of pure gold is swaged and burnished to the tooth, forming a V-shaped receptacle for the porcelain; through this backing a hole is punctured with a blunt instrument, forcing a ragged margin of metal part way into the hole in the porcelain, into which a mat of foil-gold is then packed. Removing the porcelain, the backing and the plug of gold are removed together, inverted and soldered into one mass, to which, when ready, the porcelain is cemented. By this method, the porcelain not being subjected to heat, is not changed in color nor weakened by checks, and in case of subsequent fracture, a single broken facing is easily removed and replaced.

Dr. F. W. Bliss, at the Midwinter Fair Dental Congress,* described his improvement upon the Downey crown. A band and cap of platinum and a pin of iridio-platinum wire are all soldered together with pure gold before fusing on the Downey porcelain body, the strength of this crown depending on the metal; whereas the Downey crown depends largely on the porcelain for its strength.

THE CORRECTION OF INJURY RESULTING FROM EXTRACTION.

In a paper read before the New York Odontological Society,† Dr. S. E. Davenport makes an urgent appeal to all who have the advancement of their profession at heart, not to advise the extraction of teeth for the purpose of regulating, without *first taking casts*, so that the relations existing between the lingual and palatal surfaces of the teeth may be observed; this occlusion of the inner surfaces frequently having an important bearing upon the opinion formed and the advice given; the casts serve also as a most important basis for intelligent study later. He says: "The rule to always take casts and study them before advising extraction would not only serve to teach the dentist many principles, but through his greater knowledge of Nature's laws, would cause him to be less frequently a murderer

* Dental Headlight, Jan'y-March, 1895, (reprint from Pacific Coast Dentist).
† International Dental Journal, February, 1895.

of the dental organs." Any examination of the natural teeth alone must be necessarily too superficial to serve as a basis for the condemnation of teeth, as the inner surfaces of the teeth must also be studied when the jaws are closed. This can only be done from perfect casts, properly hinged."

### TWO SIMPLE METHODS OF TREATMENT OF FRACTURES OF THE LOWER JAW.

Dr. Thos. L. Gilmer, in a paper read before the Wisconsin State Dental Society,* said that every dentist might reasonably anticipate being called upon, either in consultation or alone, to treat fractures of the jaws, and it should be his aim to be prepared either to take sole charge of the case or to give valuable assistance to the surgeon in charge. He gave two simple methods, original with himself, by which the majority of fractures of the lower jaw may be successfully treated.

In the first method, soft iron wires of sufficient length are placed about the cervical portion of a number of teeth in each jaw, anterior and posterior to the fracture. The wires are twisted to fit the teeth closely, the teeth of both jaws brought into articulation, and the ends of the wires secured together, bringing the lower jaw firmly against the upper, a bandage being used, if necessary, to overcome muscular contraction. If no teeth have been lost, one might be sacrificed in order to feed the patient by means of a syringe with sufficiently large nozzle.

In the other method, an impression is taken of the teeth in both jaws, and casts made. If necessary, the cast of the lower jaw may be sawed in two on the line of fracture, and so reconstructed as to secure correct articulation with the occlusal surfaces of the teeth in the upper jaw. Reproduce the cast of the lower jaw in Melotte's metal, and swage a plate to cover several teeth anterior and posterior to the fracture, and extending down at least two-thirds of the length of the teeth. Cement the splint to the teeth with phosphate of zinc. This is cleanly, easily made, and permits the mouth to be opened and closed at will.

* Dental Review, January 15, 1895.

[*To be Continued.*]

